# Characterizing Gene Copy Number of Heat Shock Protein Gene Families in the Emerald Rockcod, *Trematomus bernacchii*

**DOI:** 10.3390/genes11080867

**Published:** 2020-07-31

**Authors:** Anthony D. Tercero, Sean P. Place

**Affiliations:** Department of Biology, Sonoma State University, 1801 E. Cotati Ave, Rohnert Park, CA 94928, USA; terceroa@sonoma.edu

**Keywords:** notothenioid, heat shock proteins, cellular stress, molecular chaperones, gene duplication

## Abstract

The suborder Notothenioidae is comprised of Antarctic fishes, several of which have lost their ability to rapidly upregulate heat shock proteins in response to thermal stress, instead adopting a pattern of expression resembling constitutive genes. Given the cold-denaturing effect that sub-zero waters have on proteins, evolution in the Southern Ocean has likely selected for increased expression of molecular chaperones. These selective pressures may have also enabled retention of gene duplicates, bolstering quantitative output of cytosolic heat shock proteins (HSPs). Given that newly duplicated genes are under more relaxed selection, it is plausible that gene duplication enabled altered regulation of such highly conserved genes. To test for evidence of gene duplication, copy number of various isoforms within major heat shock gene families were characterized via qPCR and compared between the Antarctic notothen, *Trematomus bernacchii*, which lost the inducible heat shock response, and the non-Antarctic notothen, *Notothenia angustata,* which maintains an inducible heat shock response. The results indicate duplication of isoforms within the *hsp*70 and *hsp*40 super families have occurred in the genome of *T. bernacchii*. The findings suggest gene duplications may have been critical in maintaining protein folding efficiency in the sub-zero waters and provided an evolutionary mechanism of alternative regulation of these conserved gene families.

## 1. Introduction

The unique thermal and geographic isolation of Antarctica creates some of the coldest, most stable waters in the world’s oceans. The formation of the South Tasman Rise and the opening of the Drake Passage led to the movement of water which feeds what is now known as the Antarctic Circumpolar Current (ACC) [1]. The creation of the ACC limited polar heat convergence and resulted in the gradual cooling of sea surface temperatures around Antarctica [2]. What remains is a continent and ocean that has been thermally isolated for millions of years and is currently characterized by sub-zero waters which have been a major driver of variance and evolution of organisms that inhabit the Southern Ocean [3,4]. During the time of the gradual thermal isolation of Antarctica, it is believed that a perciform notothenioid ancestor radiated into the Southern Ocean, adapting to the gradually cooling waters and leading to the modern day Notothenioid suborder, the most diverse and dominant suborder of fishes in the Southern Ocean. Currently, Antarctic notothenioid fishes are comprised of over 120 species and represent one of the most robust examples of adaptive radiation in a marine environment [5,6].

Broadly speaking, adaptive radiation results from a single ancestor radiating into a new environment and through the acquisition of key evolutionary innovations, colonization of new habitats, and/or extinction of antagonists, the newly radiated organism can differentiate into an array of morphological and ecologically diverse species [7,8,9]. Adaptive gene duplication typically accompanies adaptive radiation and is an evolutionary process in which duplicated genes often provide fitness advantages. To this point, adaptive gene duplication has been observed throughout all taxa, including those in the Antarctic, and is overwhelmingly biased toward specific gene functions, such as genes associated with various stress-induced pathways [10,11,12]. Other examples of Antarctic-specific gene duplication include genes involved in the heat shock response (HSR) [13,14]. 

The heat shock response (HSR) is a highly conserved cellular mechanism that plays an important role in maintaining protein homeostasis in stressful environments. Heat shock proteins (HSPs), also known as chaperones, are upregulated during the HSR and function by binding to and interacting with misfolded client proteins [15,16,17]. Molecular chaperones come in two major forms based on regulation of their expression: inducible and constitutive. Constitutive chaperone expression is generally responsible for assisting with de novo protein folding of nascent proteins, whereas inducible chaperones are upregulated over relatively short-time frames in response to more immediate disruptions to protein homeostasis [15,16,17]. Despite being conserved in nearly every organism studied to date, previous studies analyzing the thermal stress response of Antarctic notothenioids, such as *Trematomus bernacchii,* have suggested that the HSR has been altered in these fishes and that major HSP gene families lack the ability to be rapidly upregulated [18,19,20,21]. At the level of the transcript, it appears as though this alteration of the HSR may be related to a change in the regulatory region that has led to the constitutive or continual expression for at least some of these ancestrally inducible genes [20,21,22,23]. Our comparator species used in the study, a closely related non-Antarctic notothenioid, *Notothenia angustata,* also has evolutionary origin in the Southern Ocean, but unlike *T. bernacchii* has retained its inducible HSR since radiating into the modern day cold-temperate oceans near New Zealand [24]. The co-opting of this constitutive expression pattern for typically inducible genes previously observed in multiple Antarctic notothenioid species is thought to be driven in part by an increased need for chaperoning activity given the denaturing effects sub-zero temperatures can have on proteins [25].

A likely possibility for functional changes resulting in the loss of inducibility of HSPs in *T. bernacchii* may lie in alterations in the architecture of the genome via gene duplication events. Unlike point mutations, gene duplications occur at rates that can be magnitudes higher and often accompany adaptive radiation events [26,27]. However, in order for both copies of a newly duplicated gene to be retained, they must both be under some positive selection, and, for HSP genes in Antarctic species, the need to buffer against the perturbing effects of sub-zero water temperatures may have provided the necessary selective pressures. Given that newly duplicated genes are under somewhat more relaxed selective pressures [12,28,29], it is plausible that retention of gene duplicates enabled alterations in the regulatory regions of the inducible HSP genes, enabling de novo regulation of such highly conserved gene products. This potential evolutionary mechanism has been further supported by a recent study performed by Bogan and Place [30], which provides evidence that notothens within the Antarctic clade, including trematomids closely related to *T. bernacchii,* displayed accelerated evolution and reduced purifying selection at promoter regions for several HSP genes.

Given the previous studies that have demonstrated gene duplications in gene families related to protein folding in Antarctic notothenioids, accelerated evolutionary rates at HSP promoter regions in several species of cryonotothens, and the interruption of the canonical HSR in *T. bernacchii*, the major objective of this study was to perform qPCR SYBR assays to test for evidence of gene duplication in three major HSP gene families (*hsp*40, *hsp*70, and *hsp*90) that display elevated expression under ambient seawater conditions and compare gene copy number to the cold-temperate notothenioid, *Notothenia angustata*.

## 2. Materials and Methods

### 2.1. Collection of Fish

Specimens of the Antarctic notothenioid, *T. bernacchii,* were collected from McMurdo Sound Antarctica using hook and line fishing through a hole drilled in the sea ice. Once collected, fish were immediately placed in aerated coolers and transported back to McMurdo Station and housed in large flow-through aquariums held at near-ambient seawater temperatures (−1.5 °C). Specimens of the non-Antarctic notothenioid, *N. angustata*, were caught using baited traps set on the substrate near Portobello Marine Laboratories (University of Otago) on the Otago Peninsula of the South Island of New Zealand. All fish were euthanized in MS-222 before tissue samples were removed and flash frozen in liquid nitrogen prior to shipment back to Sonoma State University where all samples were maintained at −80 °C. 

All organisms used in this study were housed and sacrificed according to approved animal use protocols dictated by the Institutional Animal Care and Use Committee at Sonoma State University (protocol # 2015–51, PHS assurance # N17000001).

### 2.2. DNA Extraction & QC

DNA was extracted from white muscle and gill tissues collected from *n* = 20 *T. bernacchii* and *n* = 5 *N. angustata* specimens using standard manufacturers protocol for the DNeasy^®^ Blood and Tissue Kit (QIAGEN, Hilden, Germany). Variation in sample size is due mainly to permit and population concerns for *N. angustata*. Following the DNA extraction, all samples were quantified using a Qubit^®^ 3.0 Fluorometer (Life Technologies, Singapore) and DNA integrity was checked by running extracted DNA on a 1.5% agarose gel for 60 min at 90 V.

### 2.3. Cloning and qPCR Primer Design

Target genes were identified based on previous gene expression data from *T. bernacchii* that allowed us to exclude potential pseudogenes. Additionally, several isoforms listed were also in the study performed by Bogan and Place (*hsc*71, *hspa*6, *hspa*13, *hsp*90*ab*1), which allows us to relate gene copy number data with promoter sequence evolution in those genes. It is important to note that the gene target list referenced in Table 1 is not a complete representation of the total number of genes within each selected superfamily for each study species. Furthermore, given that more atypical HSP70 family members (*hspa12a* and *hspa13*) may not share high levels of sequence similarity between species, some HSP members may be amplified in one species, but not in the other [31]. As such, we limited our target genes to only those isoforms that could be efficiently amplified in both species. Next, gene specific primers were designed for *hsp*40, *hsp*70, and *hsp*90 orthologs identified in the genome of a closely related Antarctic notothenioid, *Notothenia coriiceps,* using Primer-BLAST^®^ on the NCBI database to allow us to focus on regions that differentiate specific isoforms (Table 1).

For *hsp*70 isoform *hspa*4*b* for both *T. bernacchii and N. angustata*, a fragment of 300–500 bp was amplified from genomic DNA using primers designed from contigs obtained from an annotated transcriptome library for *T. bernacchii* [20,21]. Following PCR amplification, the gene fragments were ligated into a pCR-4 TOPO sequencing vector (Invitrogen, Waltham, MA, USA) and transformed into One Shot^®^ TOP10 chemically competent *E. coli* cells (Invitrogen) using the manufacturers recommended protocol for chemical transformation. Plasmids were isolated using the QIAprep^®^ Spin Miniprep Kit (QIAGEN) using the manufacturer’s recommended protocol and quantified using the Qubit^®^ 3.0 Fluorometer, followed by sequencing of the plasmid at MCLabs to verify the insert represented the gene of interest. Once we had successfully cloned each species-specific gene fragment, we utilized these sequences to design gene and species-specific qPCR primers for copy number determination (Table 2). Primers were designed using Primer-BLAST^®^ software (NCBI, Bethesda, MD, USA) offered in the NCBI web-interface. Subsequently, primers were subjected to in silico validation to avoid secondary structure formation at annealing sites using MFOLD v 3.5 (Washington University, St. Louis, MO, USA). Following in silico quality control, primers were tested in a standard PCR reaction using both *T. bernacchii* and *N. angustata* template to confirm the primers amplified a single band of expected product size. Primer efficiency was then determined via qPCR and specificity was further confirmed by running a melt curve analysis. All oligonucleotides were synthesized by eurofins Genomics™ (Louisville, KY, USA). 

### 2.4. Preparation of Standard Plasmids for Quantitative PCR

Currently, there are no known copy number data available for *T. bernacchii* or *N. Angustata*; therefore, we used a gene-specific plasmid for our copy number control. This approach has an added advantage as it also eliminates qPCR primer bias between species. However, previous research has suggested that overestimation of qPCR quantification can occur if uncut supercoiled plasmids are used as a standard [32]. Therefore, prior to use in qPCR assays, each copy number control plasmid was linearized with the NotI enzyme (Promega, Maddison, WI, USA). We used NEBcutter V2.0 (NEW ENGLAND Bio Labs, Ipswich, MA, USA) to verify each plasmid contained only a single cut site that occurred outside our insert. After linearization, the plasmid was purified with the QIAquick PCR Purification kit (QIAGEN, Hilden, Germany) following standard protocol and quantified with a Qubit^®^ 3.0 Fluorometer. Next, the number of copies per µL was calculated for each purified plasmid following the equation below where *X* is the amount of input plasmid DNA and *N* is the total length of the sequence (size of vector + size of insert).

(1) $$Number\;of\;Copies=\frac{Xng*6.0221\times 10^{23}\;copies/mole}{\left(N*\frac{660g}{mole.bp}\right)\times 1\times 10^{9}ng/g}$$

Lastly, a 5-fold 1:10 serial dilution series was performed, creating our working plasmid copy number control samples that ranged from 10^7^ to 10^3^ copies/µL. A new copy number control series was created, and the process repeated for each species and gene being characterized. 

### 2.5. Conducting Copy Number Determination using Quantitative PCR

Biological samples (*n* = 20 *T.b.*, *n* = 5 *N.a.*) were measured in triplicate using iTaq Universal SYBR Green Supermix with the absolute quantification parameters on a 7900 H Fast Real-Time PCR System (ABI, Foster City, CA, USA). Individual reactions (20 µL total) contained the following components: 10 µL SYBR Green iTaq, 0.5 µM F’ + 0.5 µM R’ primers, 7 µL nuclease-free water, and 1 µL template. Ninety-six-well plates were sealed and briefly centrifuged before loading into the qPCR machine and ran with the following cycling parameters: 95 °C for 5 min and 40 cycles of 95 °C for 15 s, 60 °C for 15 s, and 72 °C for 15 s. A melt curve analysis was performed after every run to ensure no contamination or non-specific amplification could be detected. For the standard curves, the Ct values were plotted against the log concentration of plasmid copy number and a linear fit was applied. In addition to verification of our amplification efficiency using the slope of the linear fit, we also determined the linearity (represented by the correlation coefficient r^2^) and the y-intercept of the line, which is required for copy number calculations. 

### 2.6. Copy Number Calculations and Determination

To evaluate the accuracy of our copy number controls, and therefore the accuracy of our copy number assay, an “Expected Copy Number vs. Calculated Copy Number” calculation was performed. For determining plasmid copy number, the following equation was used:

(2) $$Xo=E\;{\left(AMP\right)}^{Ct-Cq}$$

where *Xo* is the starting amount of copies in the reaction, *E_(AMP)_* is the amplification efficiency of the reaction as determined by the slope of the linear fit, *Ct* is the arithmetically averaged *Ct* value of the target standard, and *Cq* is the Y-intercept of the line. From this equation, we determined the “Calculated copy number” of all series within our standard dilutions and found the percent difference compared to its “Expected Copy Number”. To account for differences in primer efficiency between plasmid template DNA and genomic DNA, we also performed a correction to the *Ct* value using the “One-Point Calibration” (OPC) method [33]. OPC is performed by defining a standard containing a known amount of copies within a standard curve (N_o STANDARD_). The corrected starting copies of our gene of interest (N_o SAMPLE_) can be estimated from the cycle thresholds C_t SAMPLE_ and C_t STANDARD_ and the amplification efficiencies of E_SAMPLE_ and E_STANDARD_.

(3) $$No_{SAMPLE}=No_{STANDARD}\times \frac{E{\left(AMP\right)}^{Ct}{}_{STANDARD}}{E{\left(AMP\right)}^{CT}{}_{SAMPLE}}$$

Once the copies of our target gene were calculated for the qPCR reaction, we determined how many copies were present within a single haploid individual. Each individual template was diluted between 1 to 2 ng/µL and then quantified via Qubit fluorometer for exact concentration. The below equation was used to determine the number of genomes loaded into each qPCR reaction where *X* is the amount of genomic DNA (pg) loaded into a single well divided by the *c*-value. Recent estimates of the genome size for *T. bernacchii* suggest a *c*-value of 1.19 for this species [34,35]. It is important to note that, at the time of this study, there were no known published *c*-values available for *N. angustata*, therefore the *c*-value of a closely related notothen, *N. coriiceps* (1.13), was used [36].

(4) $$Genomes\;per\;rxn.=\frac{Xpg}{\left(C-value\right)\;}$$

Once the number of genomes was calculated, each sample reaction was normalized, and the number of copies per haploid genome was determined by dividing the total number of copies by the total number of genomes.

### 2.7. Statistical Analysis

All statistical analyses were performed using R Studio™ ver. 1.1.463 (Boston, MA, USA). The distribution of average copy number for each characterized gene from each species was tested for normality using a Shapiro–Wilk test. Copy number variation between the two species for each gene was tested for equal variance using an F test. An independent T-test was run on each gene with variance defined as nonequal or equal to measure the statistical significance between average copy number between our two species. In one instance where the distribution of copy number data was not normal (*hsp*90*b1*), a Mann–Whitney U test was used to test for significant difference in mean copy number between *T. bernacchii* and *N. angustata*. Detailed statistical reporting of isoform specific copy number variation is provided in the Supplementary Materials (Data S1).

### 2.8. Data Availability

Sequence data used to create qPCR primers and plasmid copy number standards for this study have been deposited on NCBI (Data S2). All other data can be accessed through the U.S. Antarctic Program Data Center under the following project record: https://www.usap-dc.org/view/project/p0010055.

## 3. Results

**Figure 1:**
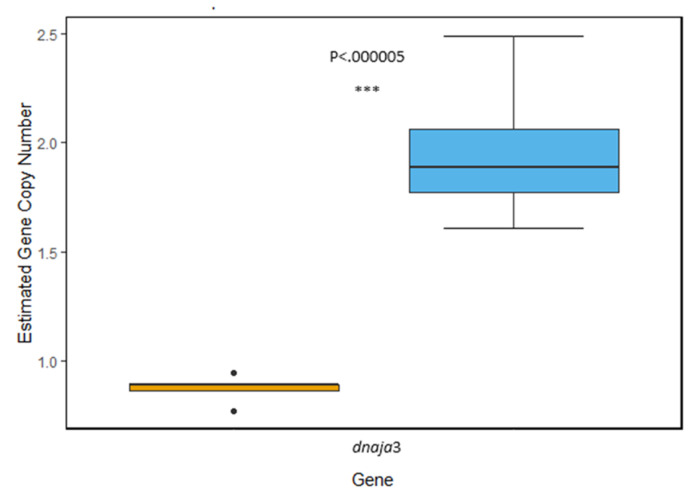
Comparison of estimated gene copy number of the *hsp*40 isoform *dnaja*3. Average copy number is estimated to be 1.92 copies per haploid genome in *T. bernacchii* (blue) and 0.875 copies per haploid genome in *N. angustata* (orange). A highly significant difference between the two means was observed (*p* < 0.00005). Box and whiskers represent quartile ranges with circles representing outliers.

*hsp40*: Within the *hsp*40 gene family, we characterized the isoform *dnaja*3 and found evidence of gene duplication in the genome of *T. bernacchii*, which contained significantly more copies than *N. angustata* (*t*_(22.554)_ = −17.505, *p* = 1.29 × 10^−14^). From our measurements, it appears *T. bernacchii* has two copies per haploid genome compared to an estimated one copy of *dnaja*3 in *N. angustata* (Figure 1). 

**Figure 2:**
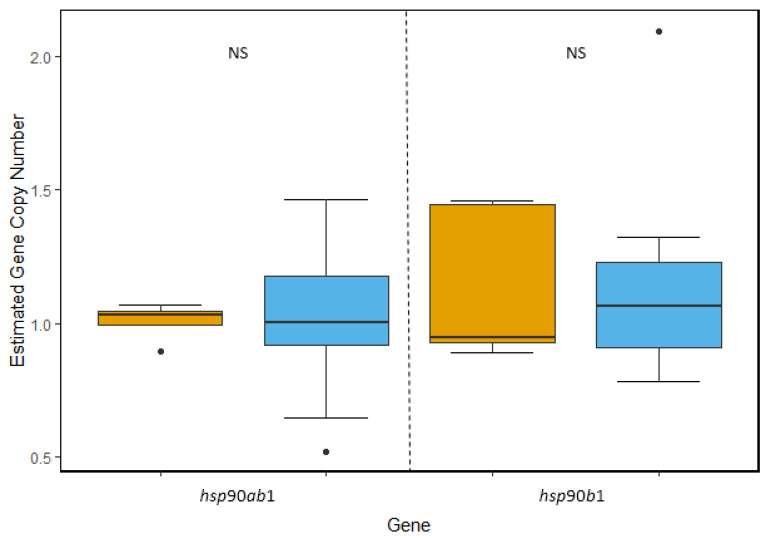
Comparison of estimated gene copy number of two isoforms within the *hsp*90 gene family. The average copy number of ancestrally constitutive isoform *hsp*90*ab1* was determined to be 1.07 copies per *T. bernacchii* genome (blue) and 1.01 copies per *N. angustata* genome (orange). Within the inducible isoform *hsp*90*b*1, *T. bernacchii* had an average of 1.1 copies and *N. angustata* had an average of 1.14 copies per haploid genome. No significant difference in average copy number was observed between species for either of the two isoforms. Box and whiskers represent quartile ranges with circles representing outliers.

*hsp90*: We targeted two different isoforms within the *hsp*90 gene family, the ancestrally constitutive isoform *hsp*90*ab*1 and the inducible isoform *hsp*90*b*1. Unlike *hsp*40, we found no evidence of gene duplication within this gene family. For *hsp90ab1*, we found no significant difference between the estimated copy number between the two species (*t*_(22.133)_ = −0.3846, *p* = 0.7042), with each species containing a single copy per genome. Similarly, we found each species had an estimated single copy per genome of *hsp*90*b*1, again with no statistically significant difference between species (*u*(w) = 47, *p* = 0.9138) (Figure 2).

**Figure 3:**
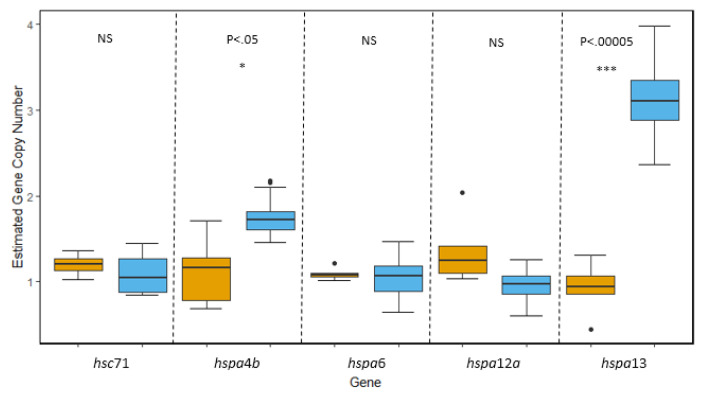
Comparisons of gene copy number of five isoforms within the *hsp70* superfamily in *T. bernacchii* (blue) and *N. angustata* (orange). For the major ancestrally constitutive isoform *hsc71,* a non-significant difference in average gene copy number was determined to be 1.1 copies in *T. bernacchii* and 1.2 copies in *N. angustata*. Isoforms *hspa*4*b* and *hspa*13 in *T. bernacchii* displayed evidence of gene duplication with an average copy number of 1.76 and 3.1 copies, respectively. Significant differences in gene copy number were observed with *N. angustata* having an average of 1.12 copies of *hspa*4*b* per genome (*p* < 0.05) and 0.92 copies of *hspa*13 per genome (*p* < 0.00005). No significant difference in gene copy number was observed for ancestrally inducible isoforms *hspa*6 and *hspa*12*a* with *T. bernacchii* carrying an average of 1.04 and 0.97 copies per genome respectively, and *N. angustata* carrying an average of 1.09 and 1.36 copies per genome, respectively. Box and whiskers represent quartile ranges with circles representing outliers.

*hsp70*: Of the molecular chaperone families, the *hsp70* family is believed to contain the largest number of paralogous genes. As such, we were able to clone and assess copy number of five different *hsp*70 isoforms from the genome of *T. bernacchii* and *N. angustata* that included both inducible and constitutively expressed variants as well as a major hsp70 co-chaperone, *hspa*4*b*. No evidence of gene duplication was observed in either species for the constitutive isoform *hsc*71 or the inducible isoforms *hspa*6 and *hspa*12*a*. However, we did find evidence that two isoforms, *hspa*4*b* and *hspa*13, have undergone duplication events within the genome of *T. bernacchii* (Figure 3). Both *hspa*4*b* and *hspa*13 showed significantly higher copy number estimates in *T. bernacchii* (*t*_(4.6063)_ = −3.3291, *p* = 2.35 × 10^−2^; *t*_(19)_ = −11.078, *p* = 9.88 × 10^−10^, respectively). For *hspa4b*, we estimated there were two copies in the haploid genome of *T. bernacchii* while *N. angustata* only possessed one copy. *hspa13* displayed the greatest amount of genomic divergence between the two species with the genome of *T. bernacchii* containing three copies of *hspa13* per haploid genome to only one copy of *hspa13* per haploid genome in *N. angustata* (Figure 3).

## 4. Discussion

The main objective of this study was to test for evidence of gene duplication of inducible and constitutive HSP genes within the genome of the Antarctic notothenioid, *T. bernacchii*. Our results show evidence of gene duplication within the *hsp*70 and *hsp*40 super families, which supports our hypothesis that duplication events may be a key mechanism that enabled relaxed purifying selection and subsequent alterations of the highly conserved regulatory regions of inducible HSP genes observed in cryonotothens closely related to *T. bernacchii* [30].

Characterization of Hsp40 isoform dnaja3 revealed some interesting results given the function and localization in some of these classes of HSPs. Although gene duplication has been observed in some members of the Hsp40 gene family in another of Antarctic notothenioid [14], it is unclear if those previously described isoforms belong to the same class of *hsp40* as *dnaja3*. Furthermore, dnaja*3* overwhelmingly localizes to the mitochondria, however some research suggests that dnaj-like proteins also play important non-mitochondrial roles, such as interacting with cytosolic Hsps [37]. Additionally, alternative splice forms of dnaja3 demonstrate longer residency time and stability in the cytosol prior to mitochondrial import. Research has suggested that the longer cytosolic transient time and the half-life of these alternative forms are explained by its interaction with cytosolic Hsc70 and other potential protein substrates [37]. Given the research suggesting non-mitochondrial roles for dnaja3 and the constitutive frontloading and increased expression of chaperones overall in Antarctic tissue, it is reasonable to assume that some alternative fates for Hsp40 may be present in *T. bernacchii* and other related species. The additional roles of *dnaja*3 may also provide insight for how these duplicates were retained in the genome of *T. bernacchii*. Given that ATP hydrolysis (protein transfer) is the rate limiting step in eukaryotic HSPs, it would seem a high molecular demand for co-chaperone activity was necessitated given the cold-denaturing effect of the extreme cold water [38]. Other means of gene duplicate retention that may be highlighted in the case of *hsp40* is gene dosage balance or dosage compensation. The gene balance hypothesis would suggest that gene duplication in one gene within a protein complex would also place positive selection for duplication within the other genes that encode for other components within that same protein complex [39]. Although we did not characterize gene copy number of *dnaja3′*s more common protein partner (hspa9) [40], some level of dosage balance and increased gene dosage may have played a role in gene duplicate retention. 

Although *hsp90* also shows an altered heat shock response and lack of rapid upregulation in response to thermal stress in *T. bernacchii* [18], we did not observe evidence of gene duplication events for this gene family. Given the specialist nature of Hsp90 [16,41,42], it is plausible that increases in gene copy number may have negative fitness implications and therefore the inability to mount a rapid inducible HSR of ancestrally inducible *hsp*90 isoforms may have resulted from other evolutionary mechanisms. Additionally, although previous studies have not observed the rapid upregulation of these genes during short-term cellular stress in notothenioid fish [18,22,23], there is some evidence that suggests the inducible isoform of *hsp*90 is still responsive to long-term thermal stress, suggesting notothenioid fish may have retained some of the normal regulatory control of this gene family [23]. The slight evolutionary conservation of hsp90 expression may also explain why previous research examining *hsp*90 showed lower proportions of accelerated evolution in Antarctic notothens [30].

The gene duplication results detected in isoform *hspa*4*b* is noteworthy. Although hspa4b is highly related to the hsp70 class of chaperones, the isoform itself encodes for Hsp70-related Hsp110 co-chaperones [43]. Although it seems that members of these co-chaperone proteins do play some role in direct protein folding, arguably the most important role observed in hspa4b is as a nucleotide exchange factor (NEF) for other cytosolic Hsp70s [44]. Similar to how Hsp40 functions, Hsp110 co-chaperones can increase the rate-limiting step of eukaryotic HSPs, stimulating dissociation of ADP after ATP hydrolysis, which allows for a new round of binding interactions between Hsp70 and its client protein [44]. These gene duplication results in tandem with the observed gene duplication of *hspa*13 and the overall increase in cytosolic Hsps observed in Antarctic fish may further suggest a mechanism of gene duplicate retention that was previously described by the dosage balance hypothesis [39]. As a consequence to gene duplicate retention and relaxed purifying selection, increased copy number could have then facilitated divergence in the cis-regulatory sequences of these genes, further canalizing the constitutive expression of ancestrally inducible HSPs and allowing for the proactive “front-loading” of chaperones for more efficient protein folding in such a demanding environment. Evidence of relaxed purifying selection of promoter regions of HSPs has been reported in notothenioids, including members within the *Trematomus* genus in which *T. bernacchii* belongs [30]. Of the HSP genes characterized in that study, promoter regions for 9 of the 10 HSP genes displayed evidence of accelerated mutation, however, we were only able to generate evidence of duplication in one of those *hsp70* isoforms*, hspa*13. These data suggest that, while the accelerated mutational rates in these promoter regions is not the result of the gene duplication, the duplication events described here, along with the relaxed selection observed for HSP promoters, may have been key elements in the eventual change in regulation of specific inducible isoforms in *T. bernacchii*. Further examination of the contributions specific isoforms make with respect to the overall cellular levels of HSPs will be critical to deciphering the potential role gene duplication has played in the altered HSR of notothenioid fishes.

Antarctic notothenioids such as *T. bernacchii* have developed multiple adaptations that have permitted fitness in such an extreme cold environment. Studies looking into the cold-denaturing effects of sub-zero temperatures suggest that *T. bernacchii* may have evolved in an environment that is continually disruptive to protein homeostasis, which, in turn, has potentially selected for the constitutive expression and front-loading of ancestrally inducible chaperones. Provided the highly conserved nature of these gene families, it is likely that the observed duplication of ancestrally inducible genes represents a source of genetic novelty to enable rapid evolution in such a demanding environment.

## Figures and Tables

**Table 1:** PCR primers used for cloning. In all instances, each primer set works on both *T. bernacchii* (*T.b.*) and *N. angustata* (*N.a.*) genomic DNA.

Species Specificity	Gene Target	F’ Primer Sequence	R’ Primer Sequence
*T.b./N.a.*	*hsc*71 (*hsp*70)	5-TGCTTCGTCAGGGTTGATAC-3	5-GAAAGACATCAGCGACAACAAG-3
*T.b./N.a.*	*hspa*4*b* (*hsp*70)	5-CCGAGATGCCCTGTCAATAAA-3	5-TGCTGGTTCATGGAGCTATTC-3
*T.b./N.a.*	*hspa*6 (*hsp*70)	5-TCAAGTCGGGAGAACGAAAC-3	5-CTCATCTGGGTTGATGCTCTT-3
*T.b./N.a.*	*hspa*12*a* (*hsp*70)	5-CCTGCTTACAGCACTACCATA-3	5-GAGCTCCTCACAAGGAAGATAAA-3
*T.b./N.a.*	*hspa*13 (*hsp*70)	5-ACGTTGCATGTCGCTAGAGT-3	5-GGCCAGGATAACGGAACCAA-3
*T.b./N.a.*	*hsp*90*ab1* (*hsp*90)	5-GACCAAAGCCGACCTGATTA-3	5-TCTCTTCCTTCTCCTCCTTCTC-3
*T.b./N.a.*	*hsp*90*b1* (*hsp*90)	5-CAGTACGGCTGGTCTGGAAA-3	5-TCCTCTCTCCGTAGGCCTTG-3
*T.b./N.a.*	*dnaja*3 (*hsp*40)	5-GGACTAGTGGGTGTTGGATAAG-3	5-GTGTTAAAGGTGGGACAGTTTG-3

**Table 2:** Quantitative-PCR primers and amplification efficiencies E (AMP) given for genomic and plasmid DNA. Primers with * were tested with DMSO additives to reduce suspected secondary structure formation.

Species	Gene Target	F’ Sequence	R’ Sequence	E(AMP) gDNA	E(AMP) pDNA
*T.b.*	*hsc*71 (*hsp*70)	5-TCTTATTGAGTTCCTTGCCGC-3	5-CGACATTGTCCTGGTGGGAG-3	1.97	1.96
*T.b.*	*hspa*4*b* (*hsp*70)	5-GAGGCTGAAGTGAGACCTAAAG-3	5-GCAGGCTTCCACAAATTTCAT-3	1.96	1.95
*T.b.*	*hspa*6 (*hsp*70)	5-AGGGCGTCGACTTTTACACC-3	5-GGCTTTCTCCACAGGTTCCA-3	1.94	1.97
*T.b.*	*hspa*12*a* (*hsp*70)	5-AAGGCGTATCACCTCTCAGAC-3	5-TTTCTGGGGTTGTATAGAGCGA-3	1.96	2.01
*T.b.*	*hspa*13 (*hsp*70)	5-ATTACCCAGCATCCACAGGG-3	5-TGACAGCTGTGTGATGCGAAA-3	2.00	2.00
*T.b.*	*hsp*90*ab1* (*hsp*90)	5-CTTCTCCTCCTGCTTCTTCTTC-3	5-GAAGGAGTTTGATGGCAAGAAC-3	2.01	1.95
*T.b.*	*hsp*90*b1* (*hsp*90)	5-GATGACCATACGGCGTCAGA-3	5-TCCTCTCTCCGTAGGCCTTG-3	1.91	1.90
*T.b.*	*dnaja*3 (*hsp*40)	5-CAGACCCTGCAAAAGACGGA-3	5-AATGACCACGACGGACACTT-3	1.96	2.04
N.a.*	*hsc*71 (*hsp*70)	5-GCCTGTGGAAAAGGCTCTCC-3	5-CAGCAGCTTTTGGATCTTGGG-3	1.91	2.05
*N.a.*	*hspa*4*b* (*hsp*70)	5-CCTTCATTTAGCAGGCTTCCACA-3	5-CCCATCCAGGAGAGGTACAC-3	1.90	1.94
*N.a.*	*hspa*6 (*hsp*70)	5-CGAGGGCGTCGACTTTTACA-3	5-TCCATTTTGGCGTCCCTCAG-3	1.90	1.93
*N.a.*	*hspa*12*a* (*hsp*70)	5-AGGCGTATCACCTCTCAGAC-3	5-ATCTGAAGCAAAGAAGATGCAAT-3	1.90	1.90
*N.a.*	*hspa*13 (*hsp*70)	5-ACACGTCAACATTGCATGGC-3	5-ACGTTGCATGTCGCTAGAGT-3	1.94	1.92
*N.a.*	*hsp*90*ab1* (*hsp*90)	5-CATGAAAGGCCTTAGTGCCG-3	5-GATTTAACAAACCTGGGTACCATC-3	1.95	1.94
*N.a.*	*hsp*90*b1* (*hsp*90)	5-GATGACCATACGGCGTCAGA-3	5-TCCTCTCTCCGTAGGCCTTG-3	1.95	1.92
*N.a.* *	*dnaja*3 (*hsp*40)	5-ATGACCACGACGGACACTTG-3	5-CAGACCCTGCAAAAGACGGA-3	1.94	1.94

## Supplementary Materials

The following are available online at https://www.mdpi.com/2073-4425/11/8/867/s1, Data S1: Statistical Reporting of Isoform Specific Copy Number Variation, Data S2: Accession numbers for submitted sequence data.
